# Crystal structure of bis­(3-carb­oxy-1-methyl­pyrid­inium) octa­bromide

**DOI:** 10.1107/S2056989023008460

**Published:** 2023-10-03

**Authors:** Valerii Y. Sirenko, Dina D. Naumova, Irina A. Golenya, Sergiu Shova, Il’ya A. Gural’skiy

**Affiliations:** a Department of Chemistry, Taras Shevchenko National University of Kyiv, Volodymyrska St. 64/13, Kyiv 01601, Ukraine; b Department of Inorganic Polymers, "Petru Poni" Institute of Macromolecular Chemistry, Romanian Academy of Science, Aleea Grigore Ghica Voda 41A, Iasi 700487, Romania; Vienna University of Technology, Austria

**Keywords:** crystal structure, octa­bromide anion, polyhalogen ions, *N*-methyl­nicotinic acid

## Abstract

The crystal structure of the title salt, bis­(3-carb­oxy-1-methyl­pyridinium) octa­bromide, 2C_7_H_8_NO_2_^+^·Br_8_^2−^, consists of 3-carb­oxy-1-methyl­pyridinium (*N*-methyl­nicotinic acid) cations, which are stacked between relatively rare [Br_8_]^2–^ anions. The polybromide [Br_8_]^2–^ anion has point group symmetry  and can be described as being composed of two [Br_3_]^−^ anions connected with a Br_2_ mol­ecule in a *Z*-shaped manner. Contacts between neighboring octa­bromide anions ensure the creation of pseudo-polymeric chains propagating along [111]. The organic cations are located between anionic chains and are connected to each other through O—H⋯O hydrogen bonds and to the [Br_8_]^2–^ anions through π⋯Br inter­actions that induce the creation of a supra­molecular tri-periodic network. In addition, the presence of weak C—H⋯Br contacts leads to the creation of layers, which align parallel to (11  ).

## Abstract 2

The structure of the title compound is composed of rare *Z*-shaped octa­bromide anions embedded within *N*-methyl­nicotinic acid cations.

## 1. Chemical context

Polyhalide anions have been the subject of extensive studies within the past century, whereby polyiodides offer the greatest diversity of known compounds among all polyhalide anions. The first triiodide-containing crystal structure, (NH_4_)[I_3_], was determined and characterized by Mooney in 1935 (Mooney, 1935 ▸). The known anions range from the smallest possible unit, [I_3_]^−^, through multiple discrete species of the types [I_2*n*+1_]^−^, [I_2*n*+2_]^2–^, [I_2*n*+3_]^3–^ and other types from [I_3_]^−^ to [I_29_]^3–^ (Svensson & Kloo, 2003 ▸) to infinite polymeric structures (Madhu *et al.*, 2016 ▸). A significantly smaller number of polyhalide anions is known for lighter halogens. This fact is mostly associated with the higher volatility of bromine, chlorine and fluorine in comparison with iodine, and thus their tendency to loss of halogen. However, several polybromide mono- ([Br_3_]^−^, [Br_5_]^−^, [Br_7_]^−^, [Br_9_]^−^, [Br_11_]^−^) and dianions ([Br_4_]^2–^, [Br_6_]^2–^, [Br_8_]^2–^, [Br_10_]^2–^) are also known so far (Sonnenberg *et al.*, 2020 ▸).

One of the most common applications of polybromide anions is in halogenation reactions. They are typically accessible in stable solid bulk form or as liquids with no measurable vapor pressure, depending on the organic cation. Thus, they can be handled much more easily then elemental liquid bromine (Sonnenberg *et al.*, 2020 ▸). Polybromides, for example [HMIM][Br_9_] where HMIM = 1-hexyl-3-methyl­imidazolium, have also been shown to form room-temperature ionic liquids, which can potentially be applied as a liquid electrode (Haller *et al.*, 2013 ▸). Moreover, the use of the tribromide anion in the [Br_3_]^−^/Br^−^ redox pair as a mediator in dye-sensitized solar cells has been reported to be an efficient alternative to the frequently used [I_3_]^−^/I^−^ system (Kakiage *et al.*, 2013 ▸). Polybromides have also been applied in zinc/bromine redox-flow batteries (Naresh *et al.*, 2022 ▸). 
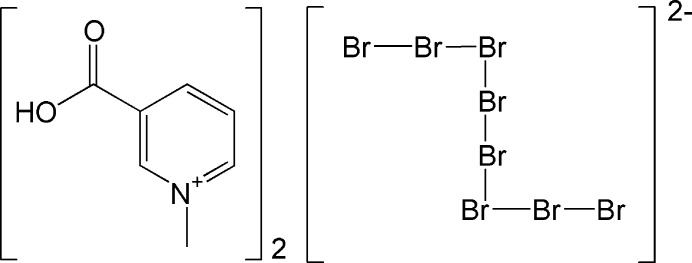


In the present communication, we report a new polybromide compound containing a *Z*-shaped octa­bromide anion, 2(C_7_H_8_NO_2_)^+^ [Br_8_]^2–^, and report its synthesis, crystal structure and Hirshfeld surface analysis.

## 2. Structural commentary

**Figure 1:**
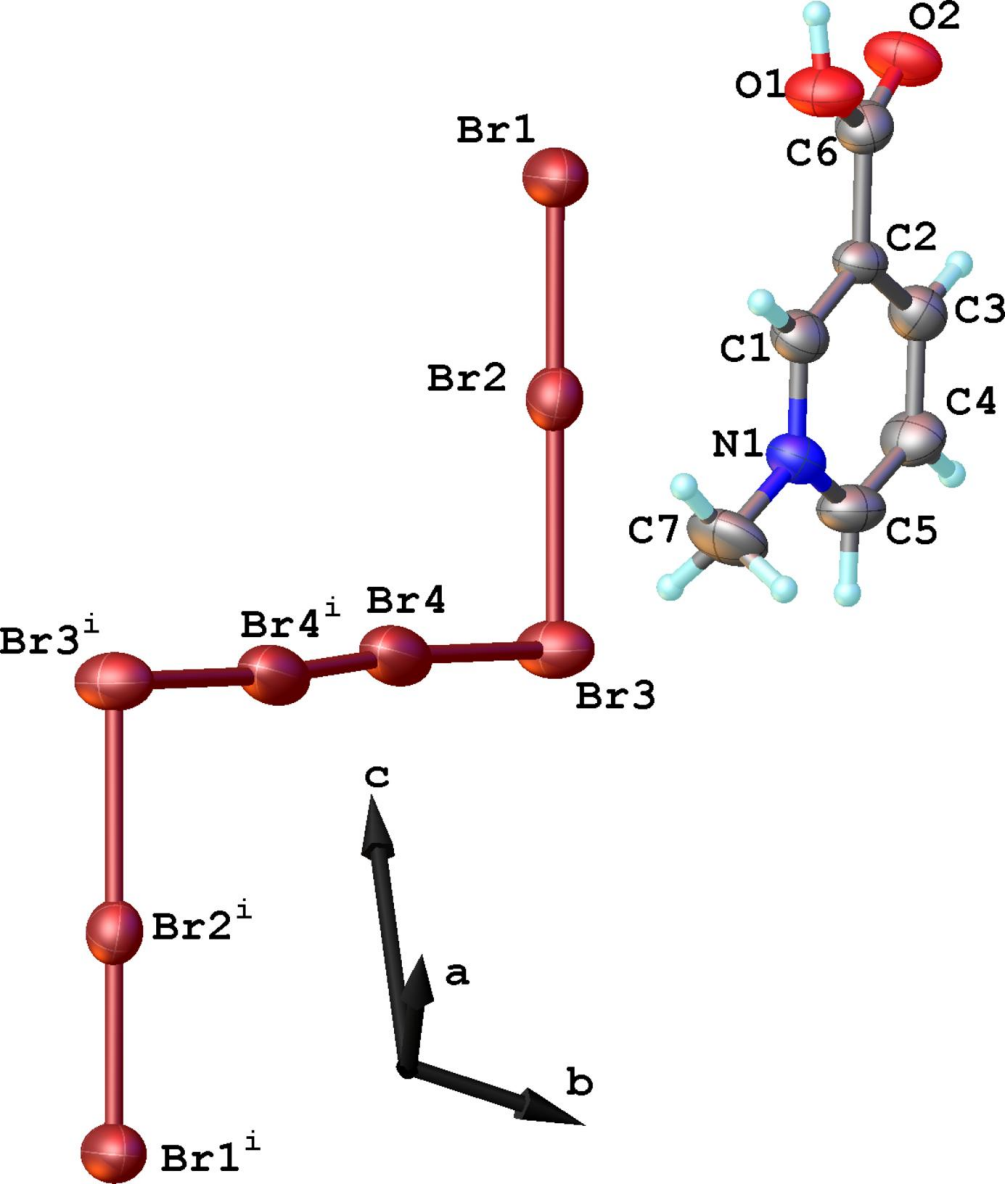
A fragment of the crystal structure of title compound showing the atom-labeling scheme. Displacement ellipsoids are drawn at the 50% probability level. [Symmetry code: (i) −*x*, −*y*, -*z.*]

**Figure 2:**
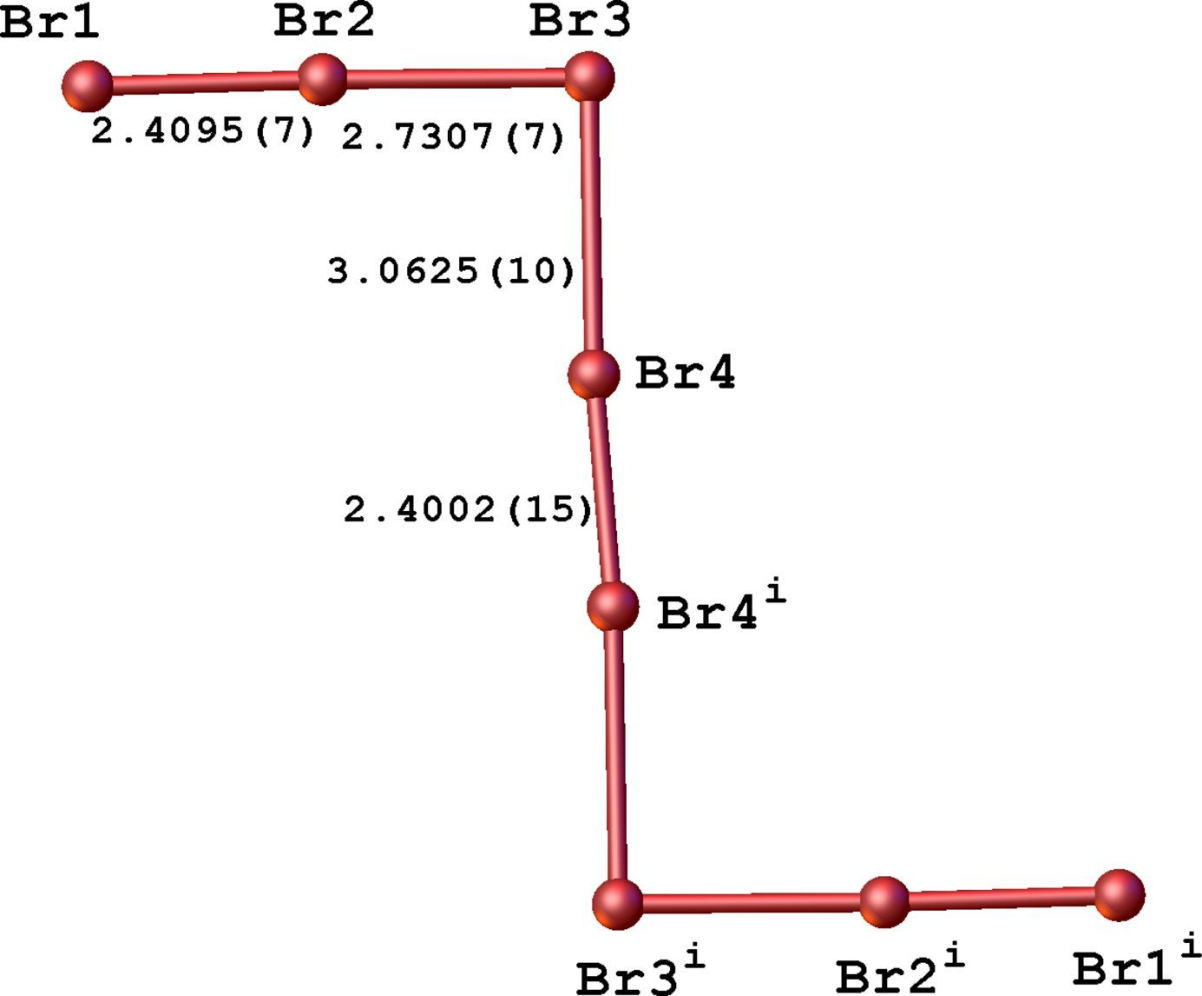
A fragment of the title compound showing the *Z*-shaped octa­bromide anion; numbers are bond lengths (in Å).

The crystal structure of the title compound consists of 3-carb­oxy-1-methyl­pyridinium (or *N*-methyl­nicotinic acid) cations separated by [Br_8_]^2–^ anions (Fig. 1 ▸). The polybromide [Br_8_]^2–^ anion can be described as two [Br_3_]^−^ moieties connected to a central Br_2_ mol­ecule in a *Z*-shaped manner (Fig. 2 ▸). The title salt has point group symmetry 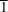 , with the inversion center located at the midpoint of the central Br_2_ mol­ecule. The Br—Br distance in the latter is 2.4002 (15) Å, which is slightly higher than 2.308 Å observed in [(Bz)(Ph)_3_P]^+^_2_[Br_8_]^2–^ where (Bz)(Ph)_3_P^+^ = benzyl­tri­phenyl­phospho­nium (Wolff *et al.*, 2011 ▸) and 2.354 Å in [*Q* ^+^]_2_[Br_8_]^2–^ where *Q* ^+^ = quinuclidinium (Robertson *et al.*, 1997 ▸). The Br1—Br2—Br3 distances in the [Br_3_]^−^ moiety of the title compound are 2.4095 (7) Å and 2.7303 (7) Å (Fig. 2 ▸). For comparison, while in [*Q* ^+^]_2_[Br_8_]^2−^ these values are similar (2.432 and 2.663 Å), in [(Bz)(Ph)_3_P]^+^_2_[Br_8_]^2−^ these bond lengths are rather equalized (2.518 and 2.498 Å). The angle between the [Br_3_]^−^ and Br_2_ fragment in the title compound is 90.37 (2)°, which lies in the range between 80° and 112° observed for the [Br_8_]^2–^ anions in other octa­bromide compounds listed in the *Database survey*. The [Br_8_]^2–^ anion in the title compound is planar with the mean deviation from the best plane through the eight atoms of 0.013 Å.

## 3. Supra­molecular features

**Figure 3:**
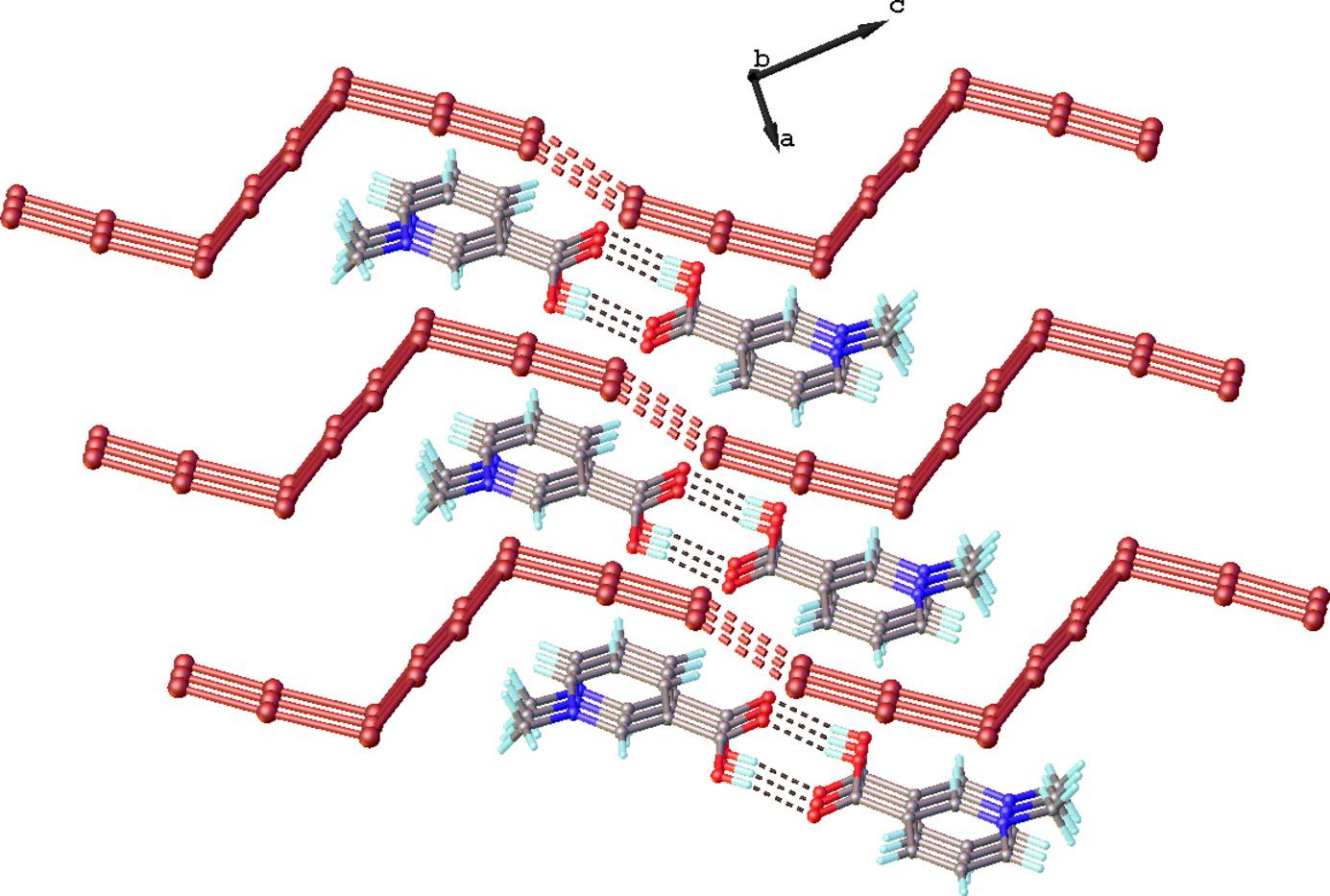
The crystal structure of the title compound in a view along the *b* axis showing infinite chains of anions. Hydrogen bonds between organic cations are shown as black dashed lines. Br⋯Br contacts between [Br_8_]^2–^ anions are shown as red dashed lines.

**Figure 4:**
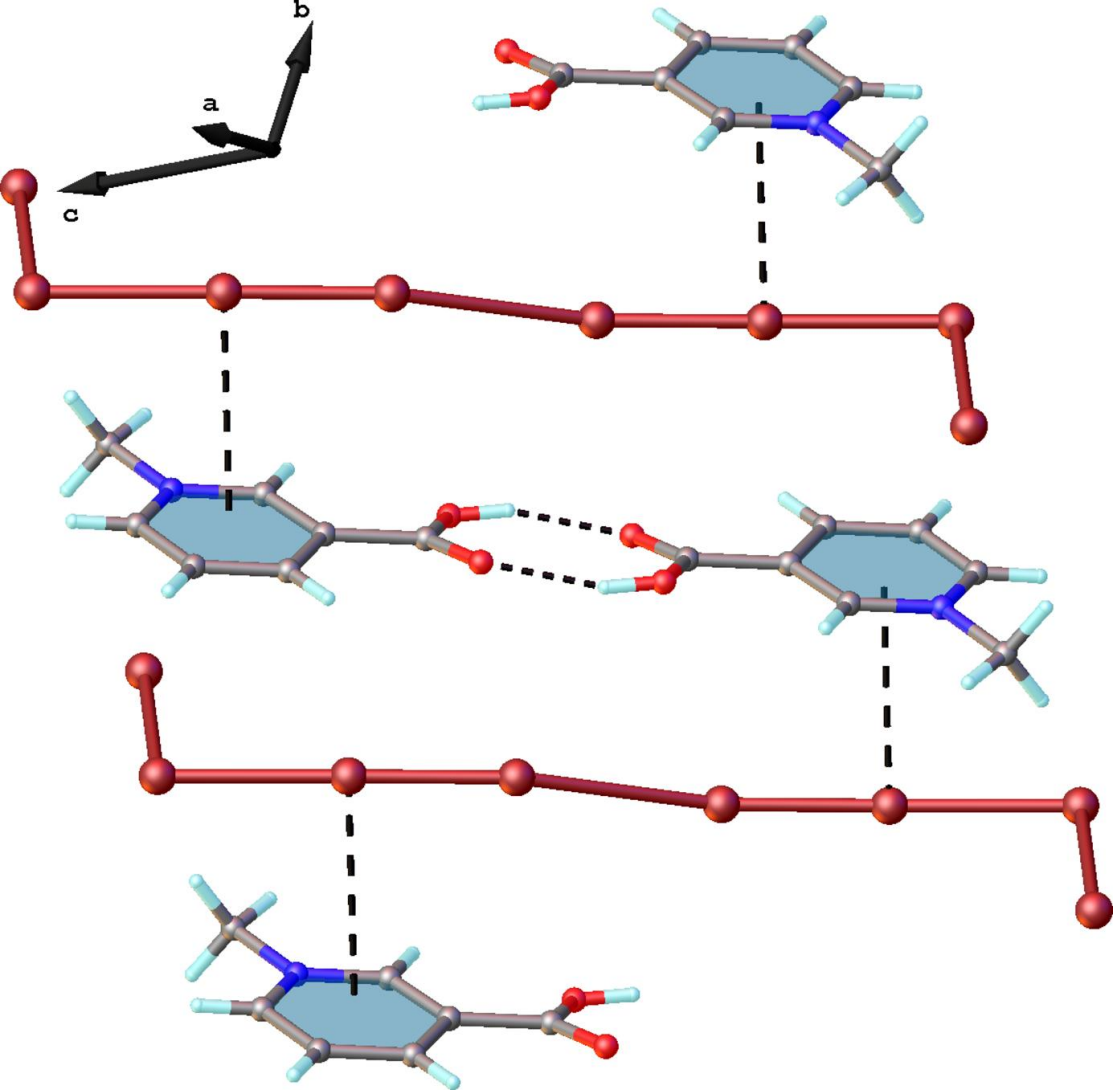
The π⋯anion inter­actions in the title compound.

The Br1⋯Br1(−*x* + 1, −*y* + 1, −*z* + 1) distance between neighboring [Br_8_]^2–^ anions is 3.1813 (12) Å, which is smaller than the sum of van der Waal radii of 3.7 Å. This inter­action contributes to the formation of infinite supra­molecular chains propagating along [111] (Fig. 3 ▸). The organic cations are located between anionic chains and are connected with [Br_8_]^2–^ through π⋯Br inter­actions [with a centroid⋯Br distance of 3.5577 (18) Å] into a supra­molecular tri-periodic framework (Fig. 4 ▸). Neighboring cations of *N*-methyl­nicotinic acid are hydrogen-bonded with each other (Fig. 3 ▸, Table 1 ▸). In addition, the organic cations show weak C—H⋯Br contacts with the polybromide anions (Table 1 ▸) that lead to the creation of layers extending parallel to (11 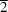 ).

## 4. Hirshfeld surface analysis

Hirshfeld surface analysis and two-dimensional fingerprint plots of the title compound were generated using *Crystal Explorer* (Spackman *et al.*, 2021 ▸).

**Figure 5:**
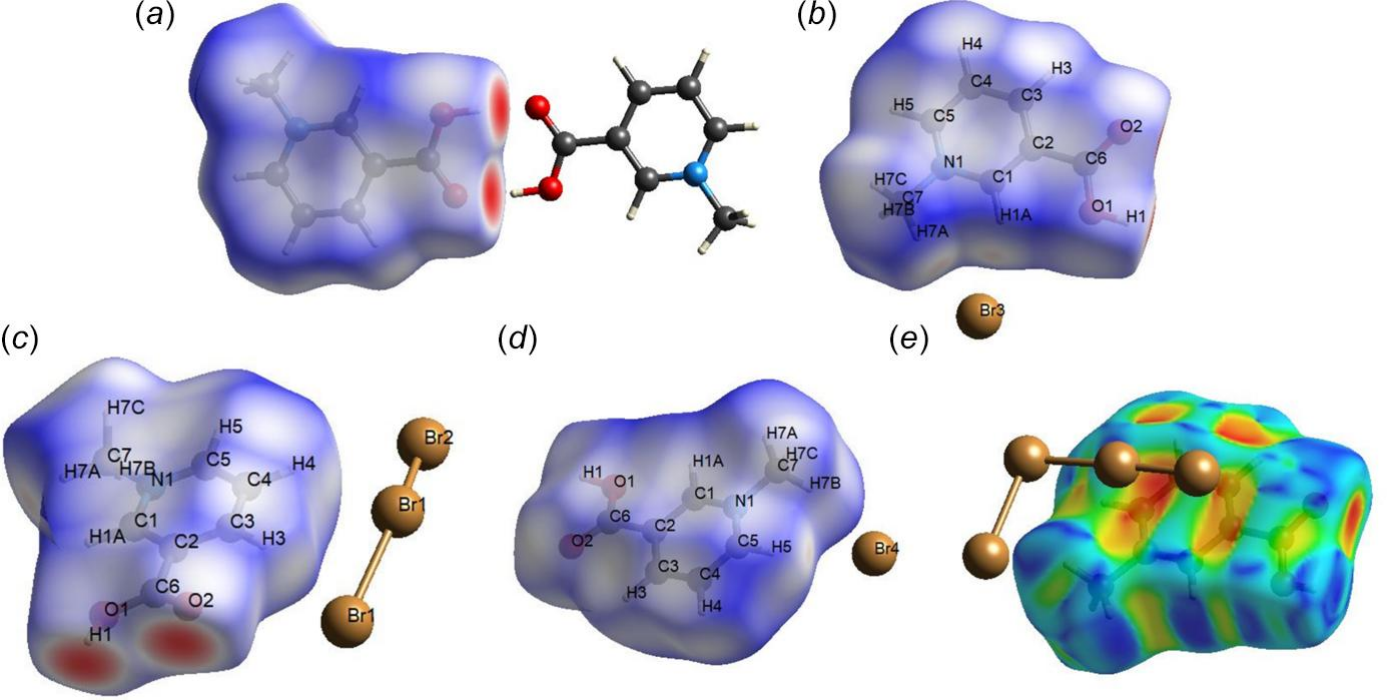
Hirshfeld surface of the 3-carb­oxy-1-methyl­pyridinium cation plotted over *d*_norm_ (*a*–*d*) or shape index (*e*). The neighboring atoms are shown in ball-and-stick mode for clarity. The surface regions with the strongest inter­molecular inter­actions are shown in red.

**Figure 6:**
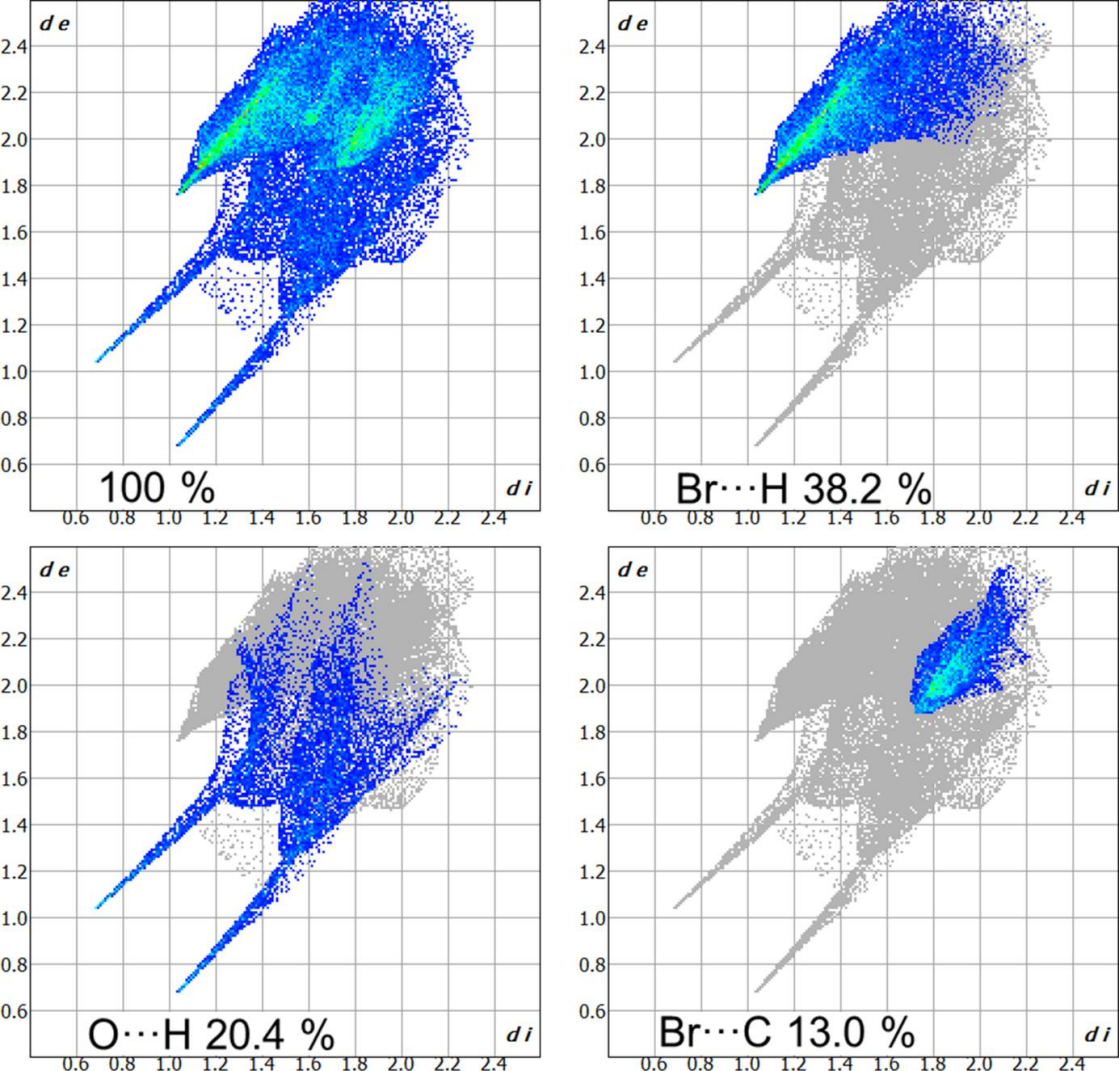
Hirshfeld surface fingerprint plot for 3-carb­oxy-1-methyl­pyridinium showing overall (100%), Br⋯H, O⋯H and Br⋯C contributions. The *d*_e_ and *d*_i_ values are the distances to the closest external and inter­nal atoms, respectively, from a given point to the Hirshfeld surface.

The graphical representation of the Hirshfeld surface of the 3-carb­oxy-1-methyl­pyridinium cation reveals the presence of a rather strong O—H⋯O hydrogen bond with a neighboring organic cation, as shown in bright red (*d*_norm_ plot, Fig. 5 ▸ *a*), and the presence of weak C—H⋯Br contacts between the organic cation and the octa­bromide anion (*d*_norm_ plot, Fig. 5 ▸ *b-d*) as well as π⋯Br inter­actions between the 3-carb­oxy-1-methyl­pyridinium and the fragment of polybromide anions located above the aromatic ring (shape-index plot, Fig. 5 ▸ *e*). The contributions of selected weak inter­actions to the crystal packing are shown as two-dimensional Hirshfeld surface fingerprint plots in Fig. 6 ▸. The strongest contribution is from Br⋯H inter­actions (38.2%) with the next major contributions from O⋯H (20.4%) and Br⋯C (13.0%).

**Figure 7:**
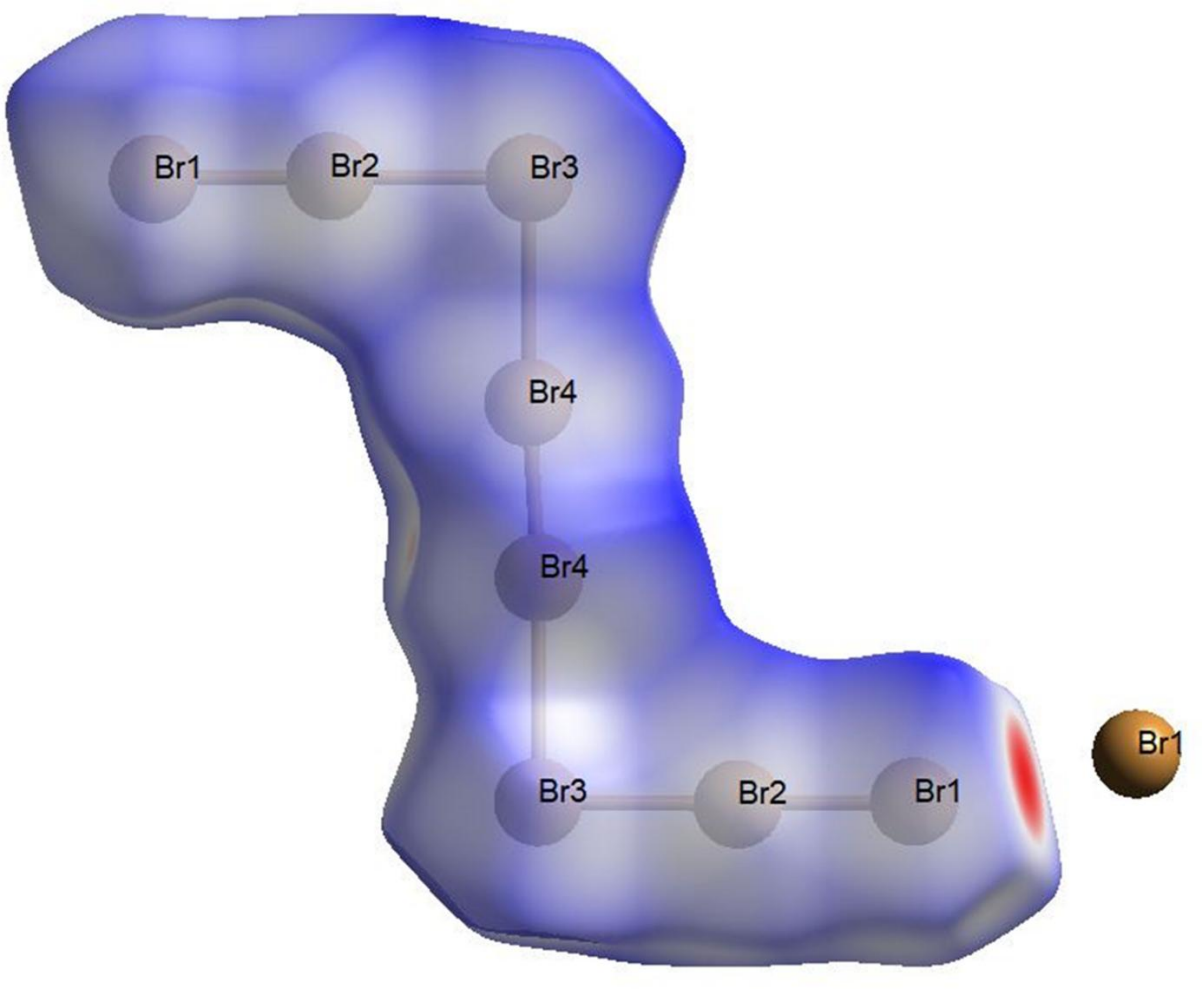
Hirshfeld surface of the octa­bromide anion plotted over *d*_norm_. The surface regions with the strongest inter­molecular inter­actions are shown in red.

**Figure 8:**
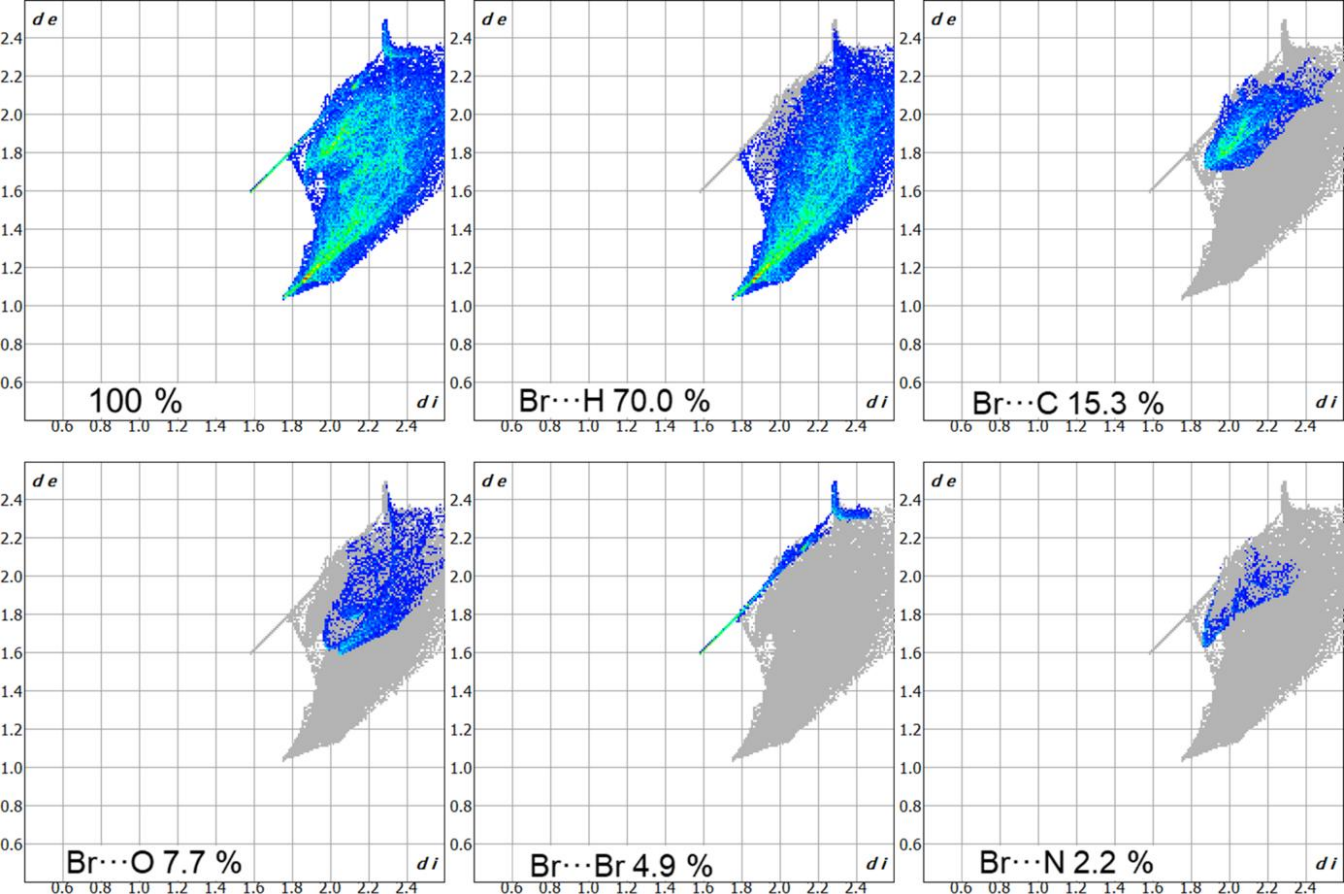
Hirshfeld surface fingerprint plot for octa­bromide anion showing overall (100%), Br⋯H, Br⋯C, Br⋯O, Br⋯Br and Br⋯N contributions.

The graphical representation of the Hirshfeld surface of the octa­bromide anion is given in Fig. 7 ▸ (*d*_norm_ plot). The most prominent inter­action is observed with a neighboring [Br_8_]^2–^ anion and is shown in red. Observed contacts with the organic cation are significantly weaker and are shown in colors from light pink to white. The fingerprint plots for the octa­bromide anion are given in Fig. 8 ▸. Here the highest contributions are observed for Br⋯H (70.0%) and Br⋯C (15.3%) contacts. Other types of inter­action make significantly smaller contribution to the crystal packing, *viz*. Br⋯O (7.7%), Br⋯Br (4.9%) and Br⋯N (2.2%).

## 5. Database survey

A search of the tribromide moiety in the Cambridge Crystal Database (CSD version 5.43, last update March 2022; Groom *et al.*, 2016 ▸) revealed 327 crystal structures, while only 28 of them containing four Br atoms connected in a row. The closest analogues to the title compound containing *Z*-shaped octa­bromide anions were found to be REKBAK (Robertson *et al.*, 1997 ▸), ICOVUS (Fromm *et al.*, 2006 ▸), RAQGIB (Wolff *et al.*, 2011 ▸) and PAQSAE (Sonnenberg *et al.*, 2017 ▸).

## 6. Synthesis and crystallization

0.5 mmol of *N*-methyl­nicotinamide was mixed with 2 ml of HBr (48%_wt_) and left to evaporate. After three days, red crystals appeared in the mixture. They were separated and kept in the mother solution prior to the diffraction measurement.

## 7. Refinement

Crystal data, data collection and structure refinement details are summarized in Table 2 ▸. Aromatic H atoms were positioned geometrically and refined with riding coordinates [*U*_iso_(H) = 1.2*U*_eq_(C)]. Methyl H atoms were positioned geometrically and were allowed to ride on C atoms and rotate around the N—C bond [*U*_iso_(H) = 1.5*U*_eq_(C)]. The H atom of the carboxyl group was found from a difference-Fourier map and was refined with a fixed distance of *d*(O—H) = 0.85 Å and with *U*_iso_(H) = 1.5*U*_eq_(O).

## Supplementary Material

Crystal structure: contains datablock(s) I. DOI: 10.1107/S2056989023008460/wm5697sup1.cif

Structure factors: contains datablock(s) I. DOI: 10.1107/S2056989023008460/wm5697Isup2.hkl

Supporting information file. DOI: 10.1107/S2056989023008460/wm5697Isup3.cml

CCDC reference: 2297637

Additional supporting information:  crystallographic information; 3D view; checkCIF report

## Figures and Tables

**Table 1:** Hydrogen-bond geometry (Å, °)

*D*—H⋯*A*	*D*—H	H⋯*A*	*D*⋯*A*	*D*—H⋯*A*
O1—H1⋯O2^i^	0.80 (7)	1.89 (7)	2.668 (5)	164 (7)
C1—H1A⋯Br3^ii^	0.93	2.96	3.838 (5)	158
C5—H5⋯Br4^iii^	0.93	2.99	3.881 (5)	160
C7—H7A⋯Br3^ii^	0.96	2.92	3.857 (6)	166

Symmetry codes: (i) 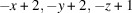 ; (ii) 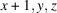 ; (iii) 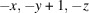 .

**Table 2:** Experimental details

Crystal data	
Chemical formula	2C_7_H_8_NO_2_^+^·Br_8_^2−^
*M*_r_	915.52
Crystal system, space group	Triclinic, *P* 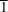
Temperature (K)	293
*a*, *b*, *c* (Å)	6.8537 (5), 7.0873 (6), 14.5145 (6)
α, β, γ (°)	95.746 (5), 91.156 (4), 115.002 (7)
*V* (Å^3^)	634.23 (8)
*Z*	1
Radiation type	Mo *K*α
μ (mm^−1^)	12.67
Crystal size (mm)	0.17 × 0.11 × 0.06

Data collection	
Diffractometer	Xcalibur, Eos
Absorption correction	Multi-scan (*CrysAlis PRO*; Rigaku OD, 2021 ▸)
*T*_min_, *T*_max_	0.458, 1.000
No. of measured, independent and observed [*I* ≥ 2u(*I*)] reflections	9457, 3008, 1848
*R*_int_	0.048
(sin θ/λ)_max_ (Å^−1^)	0.689

Refinement	
*R*[*F* ^2^ > 2σ(*F* ^2^)], *wR*(*F* ^2^), *S*	0.047, 0.075, 1.02
No. of reflections	3008
No. of parameters	132
No. of restraints	1
H-atom treatment	H atoms treated by a mixture of independent and constrained refinement
Δρ_max_, Δρ_min_ (e Å^−3^)	1.38, −1.32

Computer programs: *CrysAlis PRO* (Rigaku OD, 2021 ▸), *SHELXT* (Sheldrick, 2015 ▸), *OLEX2.refine* (Bourhis *et al.*, 2015 ▸) and *OLEX2* (Dolomanov *et al.*, 2009 ▸).

## supplementary crystallographic information

### Crystal data

**Table:**

2C_7_H_8_NO_2_^+^·Br_8_^2^^−^	*Z* = 1
*M**_r_* = 915.52	*F*(000) = 424.594
Triclinic, *P*1	*D*_x_ = 2.397 Mg m^−^^3^
*a* = 6.8537 (5) Å	Mo *K*α radiation, λ = 0.71073 Å
*b* = 7.0873 (6) Å	Cell parameters from 2335 reflections
*c* = 14.5145 (6) Å	θ = 3.2–23.7°
α = 95.746 (5)°	µ = 12.67 mm^−^^1^
β = 91.156 (4)°	*T* = 293 K
γ = 115.002 (7)°	Block, light red
*V* = 634.23 (8) Å^3^	0.17 × 0.11 × 0.06 mm

### Data collection

**Table:**

Xcalibur, Eos diffractometer	1848 reflections with *I*≥ 2u(*I*)
Detector resolution: 16.1593 pixels mm^-1^	*R*_int_ = 0.048
ω scans	θ_max_ = 29.3°, θ_min_ = 2.8°
Absorption correction: multi-scan (CrysAlisPro; Rigaku OD, 2021)	*h* = −8→9
*T*_min_ = 0.458, *T*_max_ = 1.000	*k* = −9→9
9457 measured reflections	*l* = −19→19
3008 independent reflections	

### Refinement

**Table:**

Refinement on *F*^2^	13 constraints
Least-squares matrix: full	H atoms treated by a mixture of independent and constrained refinement
*R*[*F*^2^ > 2σ(*F*^2^)] = 0.047	*w* = 1/[σ^2^(*F*_o_^2^) + (0.0186*P*)^2^] where *P* = (*F*_o_^2^ + 2*F*_c_^2^)/3
*wR*(*F*^2^) = 0.075	(Δ/σ)_max_ = −0.001
*S* = 1.02	Δρ_max_ = 1.38 e Å^−^^3^
3008 reflections	Δρ_min_ = −1.32 e Å^−^^3^
132 parameters	Extinction correction: SHELXL, Fc^*^=kFc[1+0.001xFc^2^λ^3^/sin(2θ)]^-1/4^
1 restraint	Extinction coefficient: 0.0112 (5)

### Fractional atomic coordinates and isotropic or equivalent isotropic displacement parameters (Å^2^)

**Table:**

	*x*	*y*	*z*	*U*_iso_*/*U*_eq_	
Br2	0.06745 (8)	0.45917 (8)	0.30515 (3)	0.05120 (19)	
Br3	−0.18168 (9)	0.43980 (10)	0.15210 (3)	0.0628 (2)	
Br1	0.29688 (10)	0.47662 (9)	0.43679 (4)	0.0673 (2)	
Br4	−0.04755 (10)	0.12253 (10)	0.04613 (3)	0.0781 (3)	
O1	0.9137 (6)	0.8874 (6)	0.3821 (2)	0.0564 (10)	
O2	0.7895 (5)	1.0613 (5)	0.4839 (2)	0.0540 (10)	
N1	0.4595 (6)	0.8218 (6)	0.1811 (2)	0.0420 (10)	
C2	0.6297 (7)	0.9597 (7)	0.3314 (3)	0.0348 (12)	
C6	0.7873 (8)	0.9736 (8)	0.4062 (3)	0.0430 (13)	
C1	0.6035 (7)	0.8341 (7)	0.2488 (3)	0.0423 (13)	
H1a	0.6851 (7)	0.7580 (7)	0.2398 (3)	0.0507 (15)*	
C4	0.3646 (8)	1.0582 (8)	0.2730 (3)	0.0477 (13)	
H4	0.2828 (8)	1.1345 (8)	0.2802 (3)	0.0572 (16)*	
C5	0.3421 (8)	0.9306 (8)	0.1928 (3)	0.0497 (14)	
H5	0.2430 (8)	0.9193 (8)	0.1454 (3)	0.0597 (17)*	
C3	0.5093 (8)	1.0726 (8)	0.3430 (3)	0.0444 (13)	
H3	0.5259 (8)	1.1585 (8)	0.3981 (3)	0.0533 (16)*	
C7	0.4279 (8)	0.6816 (8)	0.0942 (3)	0.0660 (17)	
H7a	0.508 (4)	0.600 (4)	0.1002 (9)	0.099 (3)*	
H7b	0.2774 (10)	0.589 (4)	0.0823 (13)	0.099 (3)*	
H7c	0.477 (5)	0.7640 (9)	0.0437 (5)	0.099 (3)*	
H1	0.992 (7)	0.879 (9)	0.426 (3)	0.099 (3)*	

### Atomic displacement parameters (Å^2^)

**Table:**

	*U*^11^	*U*^22^	*U*^33^	*U*^12^	*U*^13^	*U*^23^
Br2	0.0515 (4)	0.0417 (3)	0.0592 (3)	0.0185 (3)	0.0015 (2)	0.0076 (2)
Br3	0.0686 (4)	0.0771 (5)	0.0512 (3)	0.0412 (3)	−0.0081 (3)	0.0013 (3)
Br1	0.0787 (5)	0.0529 (4)	0.0661 (4)	0.0265 (3)	−0.0223 (3)	0.0019 (3)
Br4	0.0779 (5)	0.0717 (5)	0.0592 (4)	0.0069 (4)	−0.0200 (3)	0.0140 (3)
O1	0.057 (3)	0.077 (3)	0.047 (2)	0.042 (2)	−0.0104 (17)	−0.0010 (19)
O2	0.057 (2)	0.072 (3)	0.0359 (18)	0.036 (2)	−0.0069 (15)	−0.0123 (17)
N1	0.039 (3)	0.046 (3)	0.036 (2)	0.015 (2)	−0.0041 (18)	−0.0001 (19)
C2	0.029 (3)	0.040 (3)	0.032 (2)	0.012 (2)	−0.0030 (19)	0.006 (2)
C6	0.038 (3)	0.045 (3)	0.049 (3)	0.021 (3)	−0.002 (2)	0.005 (3)
C1	0.035 (3)	0.045 (3)	0.043 (3)	0.014 (2)	−0.002 (2)	0.004 (2)
C4	0.049 (3)	0.053 (3)	0.051 (3)	0.032 (3)	−0.002 (2)	0.004 (3)
C5	0.045 (3)	0.063 (4)	0.041 (3)	0.024 (3)	−0.007 (2)	0.009 (3)
C3	0.048 (3)	0.044 (3)	0.039 (3)	0.019 (3)	0.001 (2)	0.002 (2)
C7	0.074 (4)	0.078 (4)	0.041 (3)	0.034 (3)	−0.018 (3)	−0.020 (3)

### Geometric parameters (Å, º)

**Table:**

Br2—Br3	2.7307 (7)	C2—C1	1.380 (6)
Br2—Br1	2.4095 (7)	C2—C3	1.373 (6)
Br3—Br4	3.0625 (10)	C1—H1a	0.9300
Br1—Br1^i^	3.1813 (12)	C4—H4	0.9300
Br4—Br4^ii^	2.4002 (15)	C4—C5	1.365 (6)
O1—C6	1.290 (6)	C4—C3	1.370 (6)
O1—H1	0.845 (19)	C5—H5	0.9300
O2—C6	1.228 (5)	C3—H3	0.9300
N1—C1	1.348 (5)	C7—H7a	0.9600
N1—C5	1.332 (6)	C7—H7b	0.9600
N1—C7	1.476 (5)	C7—H7c	0.9600
C2—C6	1.483 (6)		
			
Br1—Br2—Br3	178.04 (3)	H1a—C1—C2	120.0 (3)
Br4—Br3—Br2	90.37 (2)	C5—C4—H4	120.4 (3)
Br1^i^—Br1—Br2	162.42 (4)	C3—C4—H4	120.4 (3)
Br4^ii^—Br4—Br3	176.22 (3)	C3—C4—C5	119.2 (5)
H1—O1—C6	116 (4)	C4—C5—N1	121.2 (4)
C5—N1—C1	120.7 (4)	H5—C5—N1	119.4 (3)
C7—N1—C1	119.4 (4)	H5—C5—C4	119.4 (3)
C7—N1—C5	119.8 (4)	C4—C3—C2	119.8 (4)
C1—C2—C6	120.3 (5)	H3—C3—C2	120.1 (3)
C3—C2—C6	120.6 (4)	H3—C3—C4	120.1 (3)
C3—C2—C1	119.2 (4)	H7a—C7—N1	109.5
O2—C6—O1	125.2 (4)	H7b—C7—N1	109.5
C2—C6—O1	114.6 (4)	H7b—C7—H7a	109.5
C2—C6—O2	120.1 (5)	H7c—C7—N1	109.5
C2—C1—N1	120.0 (5)	H7c—C7—H7a	109.5
H1a—C1—N1	120.0 (3)	H7c—C7—H7b	109.5
			
O1—C6—C2—C1	9.9 (5)	N1—C1—C2—C6	179.8 (4)
O1—C6—C2—C3	−169.6 (4)	N1—C1—C2—C3	−0.6 (5)
O2—C6—C2—C1	−169.9 (4)	N1—C5—C4—C3	−0.7 (6)
O2—C6—C2—C3	10.6 (5)	C2—C3—C4—C5	0.3 (5)

Symmetry codes: (i) −*x*+1, −*y*+1, −*z*+1; (ii) −*x*, −*y*, −*z*.

### Hydrogen-bond geometry (Å, º)

**Table:**

*D*—H···*A*	*D*—H	H···*A*	*D*···*A*	*D*—H···*A*
O1—H1···O2^iii^	0.80 (7)	1.89 (7)	2.668 (5)	164 (7)
C1—H1A···Br3^iv^	0.93	2.96	3.838 (5)	158
C5—H5···Br4^v^	0.93	2.99	3.881 (5)	160
C7—H7A···Br3^iv^	0.96	2.92	3.857 (6)	166

Symmetry codes: (iii) −*x*+2, −*y*+2, −*z*+1; (iv) *x*+1, *y*, *z*; (v) −*x*, −*y*+1, −*z*.
